# Net use, care and repair practices following a universal distribution campaign in Mali

**DOI:** 10.1186/1475-2875-13-435

**Published:** 2014-11-18

**Authors:** Lori Leonard, Samba Diop, Seydou Doumbia, Aboubacar Sadou, Jules Mihigo, Hannah Koenker, Sara Berthe, April Monroe, Kathryn Bertram, Rachel Weber

**Affiliations:** Department of Development Sociology, Cornell University, Ithaca, NY 14853 USA; Malaria Research and Training Center, International Center for Excellence in Research, Bamako, Mali; United States Agency for International Development, Bamako, Mali; Centers for Disease Control and Prevention, 1600 Clifton Road NE, Mailstop A-06, Atlanta, GA 30333 USA; Johns Hopkins Bloomberg School of Public Health, Center for Communication Programs, 111 Market Place Suite 310, Baltimore, MD 21202 USA

**Keywords:** Mali, Long-lasting insecticide treated nets, Malaria, Net distribution campaign, Use, Care, Repair, Perceived value of nets

## Abstract

**Background:**

The Government of Mali and the President’s Malaria Initiative conducted a long-lasting, insecticidal net (LLIN) distribution campaign in April 2011 in the Sikasso region of Mali, with the aim of universal coverage, defined as one insecticide-treated net for every two persons. This study examines how households in post- and pre-campaign regions value and care for nets.

**Methods:**

The study was conducted in October 2012 in Sikasso and Kayes in the southeast and western regions of Mali, respectively. The regions were purposively selected to allow for comparison between areas that had already had a mass distribution campaign (Sikasso) and areas that had not yet had a mass distribution campaign (Kayes). Study sites and households were randomly selected. Sleeping space questionnaires and structured interviews with household heads were conducted to obtain information on net use, perceived value of free nets in relation to other malaria prevention activities, and net care and repair practices.

**Results:**

The study included 40 households, split evenly across the two regions. Forty interviews were conducted with household heads and 151 sleeping spaces were inventoried using the sleeping space questionnaire. Nets obtained through the free distribution were reported to be highly valued in comparison to other malaria prevention strategies. Overall, net ownership and use were higher among households in areas that had already experienced a mass distribution. While participants reported using and valuing these nets, care and repair practices varied.

**Conclusion:**

National net use is high in Mali, and comparatively higher in the region covered by the universal distribution campaign than in the region not yet covered. While the Government of Mali and implementing partners have made strides to ensure high net coverage, some gaps remain related to communication messaging of correct and consistent net use throughout the year, and on improving net care and repair behaviour. By focusing on these areas as well as improved access to nets, coverage and use rates should continue to increase, contributing to improvements in malaria control.

## Background

The Government of Mali aimed to achieve universal coverage of long-lasting insecticidal nets (LLINs) by 2014. Universal coverage is defined as universal access to, and use of LLINs for populations at risk of malaria where access to a net within the household is defined as having at least one net per two people [1, 2]. The Ministry of Health (MOH) aims to achieve universal coverage through two main modes of distribution: 1) mass distribution to households as part of universal coverage campaigns; and, 2) routine distribution, primarily during antenatal care visits and in the context of vaccination campaigns [1].

The mass distribution of LLINs is designed to address malaria, one of the leading causes of morbidity and mortality in Mali, particularly among pregnant women and children under the age of five years. The national Health Management Information System (HMIS) reported 2.1 million clinical cases of malaria in 2012, and noted that 42% of all outpatient visits were due to malaria [1].

In 2006, the MOH began distributing LLINs free of charge to children under the age of five, pregnant women at their first antenatal (ANC) visit, and children under the age of one with completed vaccination cards during vaccination campaigns. Starting in 2011, mass distribution campaigns were carried out in a phased approach (region by region). The universal coverage campaign began in the region of Sikasso. As of June 2012, more than 3.9 million LLINs had been distributed in four of Mali’s nine regions, and two million more nets are planned to be distributed in 2014 [1]. The 2013 preliminary Demographic and Health Survey (DHS) report notes that household ownership of at least one insecticide-treated bed net (ITN) increased from 50% in 2006 to 84% in 2012, and 70% of children under age five had slept under an ITN the previous night in 2012 compared with 27% in 2006 [3]. Despite these gains in ownership, the same 2012 DHS survey also reported an increase in malaria parasite prevalence rates from 38% in 2010 to 52% in 2012.

Mass and routine distribution strategies in Mali have worked to achieve nationally high rates of net ownership. The overall ratio of use to access, which explains to what extent nets are used when they are available in the household, was 0.91 in 2010 [4]. Despite this, not only do some nets still go unused, nets may also be inconsistently used [5–7]. Recent studies have found the following as the most commonly reported reasons for non-use of nets: discomfort of nets due to heat, the perception of low mosquito density [8–12], outdoor sleeping [13], fears of insecticide used in treated nets [10, 13] and difficulty hanging a net [10]. However, a more recent study in Senegal [14] indicates that while respondents may be aware of barriers or annoyances to ITN use, these do not in the end impede ITN use. Similarly, in Uganda, stated difficulty hanging a net was not associated with lower rates of net use [15].

The strategy of mass distribution campaigns raises a number of operational questions: how are nets used, how are they cared for and repaired, and how are they valued relative to other malaria prevention tools that are readily available? The aim of this study was to understand how families use, care for and value nets in two regions of Mali – one where a universal distribution campaign had been carried out and one where the campaign had not yet begun. An understanding of these issues is fundamental to improving and maximizing impact of net distribution activities and associated social and behaviour change communication.

## Methods

The study was carried out from 15 October to 25 October, 2012, during the high malaria transmission season, 18 months after the Sikasso campaign. Data collection took place in two regions of the country: Sikasso, located in the south, and Kayes, located in the west. These regions were selected to allow comparisons between areas where the mass distribution had been carried out (Sikasso) and where it had not yet taken place (Kayes).

In each region, the research teams selected 20 households, ten in a rural site and ten in an urban site. In order to reduce daily travel time and to maximize the time spent in each household, the rural research sites were located within 25 km of the cities of Sikasso and Kayes. For rural sites, the research team worked with the *médecin chef* (physician in chief) at the *Centre de Santé de Référence* (CSREF, or referral hospital in the city) to establish a list of villages within a 25-km radius and to randomly select one village. In Sikasso, the village of Badabala was selected, and in Kayes, Djiguidia Peuhl was selected. In each of the two villages, village chiefs and elders helped to create a list of compounds (made up of multiple family units and households) and to randomly select compounds. Within each compound, one household was randomly selected. Within urban sites, the research team created a list of *quartiers* (city neighbourhoods), with the help of the *médecin chef* at the CSREF and one *quartier* was randomly selected for the study: Wayerma II in Sikasso and Legal Segou in Kayes. Within the selected *quartier*, households were systematically sampled by selecting a starting point at random and using cluster sampling or by approximating the number of concessions in the *quartier* and selecting every nth household.

Fieldworkers spent two to three hours in each household conducting semi-structured interviews with household heads and recording information about the nets owned by the household. For each of the sleeping spaces in the household a sleeping space questionnaire was used. The structured interviews were conducted in local language and audio-recorded. They included questions about prevention methods to determine the value of mosquito nets relative to other prevention tools; barriers to net use; advantages and disadvantages of net use; and information about how nets are used in households. The interviews included two participatory exercises. In one exercise, the household head was asked to arrange ten photographs according to the value placed on prevention methods, including nets, spray, coils, and medicines. In the other, the head of household was asked to assign photographs of ten different individuals, ranging from infant to elderly and including males and females, to four different sleeping spaces, and three bed nets. The sleeping spaces included a mat on the floor, a foam mattress on the floor, and two elevated beds with mattresses and linens. The nets included a new one with no holes, one with holes and tears of various sizes and a very worn with holes and tears.

Fieldworkers completed a structured questionnaire for each sleeping space and drew a map of each household, including numbered sleeping spaces. The map was drawn to indicate the location and type of sleeping space (bed, mat on the floor, etc.). They conducted these activities with other members of the family and, when possible, with the person who used the sleeping space. Observational data were collected for all nets associated with the sleeping spaces. Information included the brand, colour, shape, general cleanliness of the net, the number and type of repairs made to the net and the number of unrepaired holes and tears.

At the end of each day the research teams reviewed the data collection forms as a group and discussed major findings. Data from the sleeping space questionnaires were entered into an Excel spread sheet from which percentages were calculated. Interviews were translated into French and transcribed by the fieldworkers. Transcripts were reviewed and themes were identified deductively.

Approval for this research was secured from the Johns Hopkins University’s Bloomberg School of Public Health’s Institutional Review Board in Baltimore, Maryland, USA and from the Ethics Committee from *Faculté de Médicine et Pharmacie d’Odonto-Stomatologie (FMPOS)* in Bamako, Mali. All participants gave informed consent prior to participation.

## Results

A total of 40 households were included in the study, evenly split across the two regions and between rural and urban areas, and a total of 151 sleeping spaces were documented.

### Valuation of nets

In three of four localities, the majority of household heads ranked free nets first as more valuable than all other items (purchased nets, mosquito coils, insecticide or spray (Yotox), electric fans, and pharmaceuticals). The exception was Légal Segou (urban Kayes), where only three of ten household heads ranked free nets first (Table 1). In Sikasso, two respondents felt that free nets were less valuable than paid nets because you could not count on receiving them during mass campaigns. These same households noted that they had not received nets during the 2011 campaign.

**Table 1:** **Value of free nets relative to other prevention tools**

Site	Ranked free nets as most valuable prevention tool	Placed higher value on free nets relative to paid nets
**Sikasso**		
Urban (Wayerma II)	6/10	8/10
Rural (Badabala)	7/10	9/10
**Kayes**		
Urban (Legal Segou)	3/10	5/10
Rural (Djiguidia Peuhl)	8/10	10/10

### Net use

Across sites, a majority of sleeping spaces had a net associated with the sleeping space, though there was a higher proportion in the Sikasso sites (94%), where a mass net distribution had taken place, compared to the Kayes sites (65%), shown in Table 2.

**Table 2:** **Sleeping spaces and net use by site**

Site	Sleeping spaces observed	Sleeping spaces with net (%)	Sleeping spaces with net suspended (%)	Sleeping spaces where net used last night (%)
**Sikasso**				
Urban (Wayerma II)	45	42/45 (93%)	33/45 (73%)	41/45 (91%)
Rural (Badabala)	32	30/32 (94%)	22/32 (69%)	21/32 (66%)
**Total**	**77**	**72/77 (94%)**	**55/77 (71%)**	**62/77 (81%)**
**Kayes**				
Urban (Legal Segou)	35	21/35 (60%)	14/35 (40%)	18/35 (51%)
Rural (Djiguidia Peuhl)	39	27/39 (69%)	16/39 (41%)	22/39 (56%)
**Total**	**74**	**48/74 (65%)**	**30/74 (41%)**	**40/74 (54%)**

While most sleeping spaces in the Sikasso sites had a net suspended above the sleeping space at the time of the interview and observation, this was not the case in the Kayes sites. In both sites, the most common reasons given for not having the net suspended were that children played in the room during the day or that the net was removed to give the family more space for daytime activities. In some cases, families reported that cords to attach the net had just broken or that the nets had recently been washed and were not suspended because they were drying.

In all four sites, nets were reported to have been used over most sleeping spaces in the previous night. Respondents reported multiple reasons for not using nets. In some cases respondents reported that they had recently washed their nets and that the nets had not been dry enough to suspend or use them the previous night.

### Advantages and disadvantages of nets

When asked to describe what they did not like about nets and any disadvantages, all but two respondents replied that there were none they could think of. A few respondents mentioned that they did not like it when nets became dirty, torn or lost insecticide; others mentioned that sometimes they were too tired or lazy to hang or wash them; however, respondents were uniformly insistent that they saw no significant disadvantages or annoyances with net use.

Pressed further on disadvantages of nets, respondents mainly responded by framing disadvantages as an issue of access: nets were not widely available, too expensive or mass campaigns were not frequent enough or were inequitably implemented, missing families. The latter response was reported only in Sikasso, where two families had not received nets during the mass campaign 18 months’ prior. A common response was that nets would be “easier to use” if there were enough of them for all their family members.

When asked what they liked about their nets, responses fell into three main categories, with most respondents citing two or more: protection from mosquito bites and malaria, protection from other biting/nuisance insects, and comfort and getting a good night’s sleep. In Sikasso three respondents noted that nets helped keep them warmer during cold season.

### Seasonal sleeping patterns

Respondents were asked whether they ever slept outdoors. The majority of respondents in Kayes said that during hot season they did sleep outdoors to avoid hot and stifling conditions indoors, with men reportedly spending more of the night outside, and women and children spending the first part of the night outside, then going inside as the temperature became cooler. In Kayes, several respondents noted that they also slept outside during the cold season, when there were fewer mosquitoes. Two respondents in Kayes (urban and rural) said that they used their nets while sleeping outside. In Sikasso, on the other hand, sleeping outside was less common, particularly in urban areas where respondents noted that they did not have walls around their compound or that it was otherwise “too risky” to sleep outside. In the rural areas, respondents said that they slept outside only during hot season, and then only until midnight, when the temperature became cool enough to return inside. In Sikasso respondents reported that men might sleep outside while women and children remained inside all year round.

Net use and outdoor sleeping appeared to be primarily associated with perceived presence of mosquitoes. The majority of respondents said that they used nets less often in periods when they perceived a reduced density of mosquitoes, mainly during hot season or during the cold season.

### Net allocation

Respondents were asked to prioritize different types of family members, ranging from a young baby to the elderly, for a limited number of nets. Nearly all respondents felt strongly that small children and pregnant women should receive nets as first priority, citing their fragility and vulnerability to malaria. The elderly were sometimes grouped into the fragile group, while others categorized them as being strong enough to resist severe illness. Young adults were most often categorized as last priority, as they were strong and healthy, and also had means of earning money to purchase nets if they wanted them. Many respondents mentioned that as heads of household or caretakers of children, it was their responsibility to make sure that their more vulnerable family members were protected from malaria by sleeping under an ITN.

### Source of nets

The nets in the study households ranged in age from new to eight years old. They were acquired from a variety of sources, and modes of acquisition varied by region and site (see Table 3). In the Sikasso sites, most of the nets were acquired during the mass distribution or during vaccination campaigns. In the rural site, pre- and post-natal visits were also important sources of nets. In the urban site, nearly 20% of respondents reported receiving a net as a gift from a family member, a friend or someone else. There was no mention of purchasing nets among Sikasso respondents.

**Table 3:** **Sources of nets in study households**

Site	Mass distribution	Childhood vaccination campaign	Maternity ward	Market	Other
Sikasso					
Urban (Wayerma II)	26 (62%)	1 (2%)	2 (5%)	5 (12%)	8 (19%)
Rural (Badabala)	8 (27%)	10 (33%)	7 (23%)	2 (7%)	3 (10%)
Kayes					
Urban (Legal Segou)		2 (10%)		13 (62%)	6 (29%)
Rural (Djiguidia Peuhl)		9 (33%)		14 (52%)	4 (15%)

In the Kayes sites, the majority of respondents reported purchasing their nets at the market or in a pharmacy. This was the most prevalent mode of acquiring nets in the urban and in the rural localities in Kayes. In interviews, respondents noted that nets were sold at varying price levels, from 2,500 CFA to 5,000 CFA (approximately US$5-10). As in Sikasso, rural residents reported receiving the net during a vaccination campaign more often than respondents in the urban sites.

### Care of nets

The average frequency of net washing across study sites was roughly once per month. However, the frequency of net washing varied widely and ranged from never washed to washed eight times a month. Fieldworkers were asked to rate the nets as “clean”, “neither clean nor dirty” or “dirty”. The largest proportion of nets in all sites were ranked as “clean” followed by “neither clean nor dirty” and finally “dirty” – a ranking given to less than 20% of the nets. The fieldworkers’ ratings of the nets were proportionally similar across the sites and are presented in Table 4.

**Table 4:** **Observed cleanliness of nets by site**

Site	Clean	Neither clean nor dirty	Dirty
Sikasso			
Urban (Wayerma II)	21 (50%)	16 (38%)	5 (12%)
Rural (Badabala)	16 (53%)	10 (33%)	4 (13%)
Kayes			
Urban (Legal Segou)	10 (48%)	7 (33%)	4 (19%)
Rural (Djiguidia Peuhl)	12 (44.5%)	12 (44.5%)	3 (11%)

Nets were washed with soap, Omo (a packaged detergent), or both. Soap was the most common first response to the question, particularly in rural sites, but many nets were washed with more than one product – usually soap and/or Omo. Two nets had also been washed with bleach. The vast majority of nets were dried outside under the sun (n = 86). Only three nets were dried inside the house, and only 11 were dried outside in the shade.

### Repair of nets

Net repair, as defined by sewing, patching or tying knots to close holes in nets, was infrequent in both sites but more frequent in Kayes than in Sikasso. This was particularly true in the rural locality, where 15 of 27 nets (56%) had been repaired at least once. In the Sikasso sites, only nine nets, or 12.5% of the nets, had ever been repaired. Fieldworkers’ observations confirmed that few nets showed any sign of repair. The nets were generally in good condition, though some nets in both the rural and the urban sites had a small number of holes (one to five) that had not been repaired. Respondents in Badabala noted that people replace nets when they get old or torn rather than repairing them. They reportedly use old nets for other purposes, such as to transport farm produce.

It is important to note that the frequency of repair does not necessarily correspond to the number of holes in the net or the general state of the net, since repairs are often made to multiple holes at one time. The last three columns in Table 5 show fieldworkers’ counts of the number of repaired holes in the nets observed and provide different perspectives on the state of the nets in the sites. The average number of observed repairs is highest in Djiguidia Peuhl (rural Kayes), where nine of the 15 ever-repaired nets were repaired more than twice, and some nets were repaired as many as 12 times. On average, nets in that site had 3.4 repaired holes, compared to less than one for the Sikasso sites. In Wayerma II (urban Sikasso) and in Badabala (rural Sikasso) a very small number of nets in extremely poor shape skewed the overall picture, as can be seen from the data on the average number of repaired holes for ever-repaired nets and the average number of repaired holes among all nets.

**Table 5:** **Frequency of net repair**

Site	Nets repaired (reported and observed)	Maximum number of repairs (reported)	Number of holes repaired (observed)	Average number of repaired holes for repaired nets (observed)	Average number of repaired holes for all nets (observed)
Sikasso					
Urban (Wayerma II)	5 (12%)	6	1-14	4.6	0.5
Rural (Badabala)	4 (13%)	2	1-7	2.5	0.3
Kayes					
Urban (Legal Segou)	6 (29%)	4	1-11	5.0	1.4
Rural (Djiguidia Peuhl)	15 (56%)	12	1-16	6.1	3.4

## Discussion

### Value

While there was some debate in the early 2000s that free bed nets are not valued or are not valued as highly as items that people purchase [16], recent behavioural economics’ experiments have shown that women who received free ITNs were not less likely to use them [17, 18]. Results from this study did not suggest that people perceived free nets to be inferior quality or value than purchased nets. The vast majority of household heads ranked free nets as more valuable than purchased nets, although the proportion was lowest in Légal Segou (urban Kayes) where free nets had not yet been distributed. Households placed a high value on free nets relative to other tools to prevent and treat malaria, including purchased nets. The (relatively) low priority given to free nets in Légal Segou might reflect the lack of such nets, and is difficult to disentangle from what is actually available to residents in that locality. In other words, respondents may confound abstract or hypothetical value with actual value in their current circumstances. Overall, free nets were reported to be highly valued relative to other prevention methods and relative to purchased nets, suggesting that there is not a sense that nets are of low quality because they are free.

### Use

The study revealed differences in net use and sources of nets across the sites. The effects of the mass distribution campaign were evident when comparing net use and the sources of nets in the two study regions. Observed rates of possession, suspension and use were higher in Sikasso than in Kayes. Respondents in Kayes were far more likely to have purchased their nets than respondents in Sikasso, who received nets free of charge during the mass distribution campaigns or through routine distribution mechanisms.

However, within Sikasso, there were also differences in net use between the rural and urban sites, with net use (though not possession) higher in the urban than in the rural site. In contrast, the preliminary 2013 Mali DHS found that net use rates were the same in rural and urban areas [3]. In addition, the source of nets differed, with those in the rural site more often reporting receiving a net as part of routine distribution mechanisms compared to those in the urban site, who more often reported receiving their nets in the course of the mass distribution campaigns. Disruptions in delivery of ITNs through antenatal and routine vaccination visits did occur in Kayes prior to the study fieldwork. It is reasonable that in Kayes, where free nets were not available, families were more motivated to purchase nets in the market to cover sleeping spaces.

### Perception of nets

The few disadvantages of nets cited in this study relate mostly to barriers to net access. Respondents stated that nets were not widely available, too expensive and that mass campaigns were not frequent enough. The advantages cited relate mostly to protection against mosquitoes and malaria and other insect nuisances and sleeping better at night. These findings mirror those found in Senegal [14] and Tanzania [19], where respondents continued to use nets despite minor annoyances, primarily for malaria prevention benefits, but also to ensure a comfortable night’s sleep. To overcome the access issues of cost and availability, Mali may consider bolstering and expanding their routine distribution efforts beyond ANC and the Expanded Programme on Immunization (EPI) and potentially offer subsidized or socially marketed nets in the retail market. That respondents reject the suggestion that nets are annoying is a positive contribution to the literature, since many studies cite the opposite: users find the nets bothersome and therefore do not use them [8]. Concerns about insecticide were not cited as a barrier to net use, and suggests that perhaps messaging around airing out one’s net has improved or that people understand and value the protection of their LLIN. Improving access and updated behaviour change messaging about the advantages of using a net are still needed in Mali; these could effectively focus on the ancillary benefits of ITN use for getting a good night’s sleep and maintaining health for individuals and the entire household.

### Seasonal sleeping

While outdoor and seasonal sleeping habits varied in Sikasso and Kayes, most respondents slept outdoors, for either part of or all night, during the hot and cold seasons when they perceived a lower mosquito density. Outdoor net use during these seasons was low among respondents. Of 22 global studies, perceived low mosquito density during these periods is one of the most common reasons for non-net use [8]. Despite the perceived lower density of mosquitoes, Mali does have year-round malaria transmission in the southern Sudano-Guinean zone, including most of Kayes region and the entire Sikasso region [1], and net use is needed every night of the year to maintain mosquito vector control and reduce transmission. These messages should be included in strategic communication messages both at the time of the distribution, whether mass or routine, and periodically throughout the year. Further research is needed to determine whether outdoor sleeping and/or other factors contribute to persistent high parasitaemia rates.

### Net allocation

The net allocation exercise rankings, where respondents prioritized infants and pregnant women for best nets, falls in line with pre-universal coverage strategies of prioritizing vulnerable populations, and echoes priorities from studies in Uganda [20] and Tanzania [21] and analyses of recent DHS and the Malaria Indicator Survey (MIS) data [22]. While many families did express concern that there were not enough nets to cover all of their family members, they felt it was important to prioritize more vulnerable family members. Reducing access barriers, as mentioned above, will help heads of household and family caretakers ensure that they have an adequate number of nets to cover their households and sustain the gains in malaria reduction.

### Care and repair

Net care and repair practices varied widely and in some cases were inconsistent with recommended practices for net care and repair. Based on an estimated lifespan of three to four years and manufacturing specifications for LLINs that nets should retain a lethal dose of insecticide after at least 20 washes, it is a general rule of thumb that nets not be washed more than every three months to reduce insecticide degradation, although this specific messaging is rarely communicated during LLIN or malaria behaviour change communication (BCC) campaigns. Some nets in this study were reportedly washed up to eight times a month. Some respondents indicated that nets were washed with detergent and hung in the sun, both practices that contribute to insecticide loss [23]. Some nets in the study had visible holes that had not yet been repaired. Nets in poor or degraded condition have been shown to be less protective against malaria [24–26], and could be a factor in non-use [27–31]. The contributions of net care and repair practices on net integrity have begun to be explored [29, 32–36], but it is not yet known whether improved care and repair can ultimately prolong net lifespan. Strategic behaviour change interventions may affect behaviours around net care and repair. In one study in The Gambia, for example, a behaviour change intervention to promote net care and repair was associated with an increase in net repairs, from 27 to 41% of holes repaired on average per net over a four-month period [32]. Study results suggest the need for clearer recommendations on care and repair practices, and increased strategic communication on net care and repair.

### Limitations

The study aim was to understand how nets are valued and treated as malaria prevention tools, and how they fit into multipronged prevention efforts. The qualitatively driven sample was designed to be large enough to identify patterns and negative cases, but was not large enough to draw statistically significant conclusions.

## Conclusions

National net use is high in Mali, and comparatively higher in the region covered by the universal distribution campaign than in the region not yet covered. While the Government of Mali and implementing partners have made strides to ensure high net coverage, some gaps remain related to communication messaging of correct and consistent net use throughout the year, and on improving net care and repair behaviours. By focusing on these areas, as well as improved access to nets, coverage and use rates should continue to increase, contributing to improvements in malaria control.
